# The effectiveness of intensity-modulated radiation therapy versus 2D-RT for the treatment of nasopharyngeal carcinoma: A systematic review and meta-analysis

**DOI:** 10.1371/journal.pone.0219611

**Published:** 2019-07-10

**Authors:** Taifeng Du, Jiefeng Xiao, Zhaolong Qiu, Kusheng Wu

**Affiliations:** Department of Preventive Medicine, Shantou University Medical College, Shantou, Guangdong Province, China; Chang Gung Memorial Hospital at Linkou, TAIWAN

## Abstract

**Background:**

At present, the management of nasopharyngeal carcinoma (NPC) is mainly based on radiotherapy, but there are many radiation delivery techniques such as intensity-modulated radiotherapy (IMRT) and 2-dimensional radiotherapy (2D-RT).

**Materials and methods:**

We searched all the eligible studies through the PubMed, Cochrane Library, Medline, and Embase. The endpoint events in meta-analysis were overall survival (OS), tumor local control including local-regional free survival (LRFS), progression-free survival (PFS), and distant metastasis-free survival (DMFS), and late toxicities.

**Results:**

A total of ten publications met the criteria and were identified through searches of the databases and references. We included 13304 patients in the meta-analysis, of whom 5212 received IMRT and 8092 were allocated to 2D-RT alone group. Compared with 2D-RT treatment, the IMRT group was associated with a better 5-year OS (OR = 1.70; 95% CI = 1.36–2.12), LRFS (OR = 2.08; 95% CI = 1.82–2.37), and PFS (OR = 1.40; 95% CI = 1.26–1.56). Additionally, the incidence of late toxicities such as late xerostomia (OR = 0.21; 95% CI = 0.09–0.51), trismus (OR = 0.16; 95% CI = 0.04–0.60), and temporal lobe neuropathy (TLN) (OR = 0.40; 95% CI = 0.24–0.67) for NPC patients in IMRT group were significantly lower than 2D-RT.

**Conclusions:**

The meta-analysis demonstrates that IMRT provides improved long-term tumor overall survival and local control including LRFS and PFS. Additionally, IMRT yields a lower incidence of late toxicities induced by irradiation in NPC patients. Compared to 2D-RT, IMRT may be an effective treatment for patients with NPC. Further intensive studies should be pursued to examine the association.

## Background

Nasopharyngeal carcinoma (NPC) is a kind of malignant tumor that occurs in epithelial cells of nasopharynx. It is relatively rare in the world, but some regions, such as southern China, have a high incidence of up to 15–50 per 100,000[1]. The proportion of differentiated nonkeratinizing or undifferentiated carcinoma (WHO types 2 or 3) patients is higher, but the keratinizing squamous cell carcinoma or WHO type 1 was relatively lower and common in Western countries[2,3]. Due to its special anatomical location which is difficult to surgical access, and highly sensitive to radiation, radiotherapy (RT) is the primary treatment modality for locally non-disseminated NPC. While patients are treated to improve overall survival and disease-free survival, reducing acute and late toxicity and improving quality of life should be considered. In fact, the side effects of treatment largely may lead institution of treatment breaks, and then prolongs the treatment time, and ultimately generate adversely affect on the overall survival[4].

The conventional two-dimensional radiotherapy (2D-RT) was used to deliver a ‘‘tumoricidal” dose by means of laterally opposed fields until the early 1990s, which can make the disease better controlled, but be likely to cause toxicity simultaneously, including xerostomia, mucositis, hearing loss and dysphagia with its attendant sequelae, such as osteoradionecrosis. The principle of 2D-RT mainly uses shrinkage field radiation technology; the targeted field is gradually shrunk or modified to deliver the required doses[5]. The main disadvantage of the technology makes normal organs and structures such as the parotid gland compromised, but the advanced intensity-modulated radiotherapy (IMRT) is the improved way to circumvent this drawback. IMRT can deliver high doses precisely while sparing of adjacent organs at risk, cause better control and less toxicity than 2D-RT[6–8].

Compared with 2D-RT, there are many researches on the potential advantages of IMRT reflecting on its better clinical outcomes and low toxicity. Some studies have shown that NPC patients receiving IMRT treatment can achieve local control and overall survival more than 90% and 80%, respectively [9,10]. A retrospective study suggested that local relapse-free survival (LRFS) was significantly higher while the NPC patients in T1 classification received IMRT comparing with 2D-RT treatment [11]. Nevertheless, similar IMRT advantages were also reached in another prospective randomized study, LRFS did not improve in the advanced patients (T3 and T4), and it could even be said to have a driven effect [12]. A dosimetric study showed that IMRT provided better parotid gland sparing in early-stage NPC and offered better tumor coverage and normal organ sparing in locally advanced NPC because of its dosimetric advantages[13].

High survival rates are vital for improving the quality of life (QoL). Many studies have shown that IMRT treatment in NPC patients was better than 2D-RT in sparing the parotid gland, improving quality of life[14,15] and the decrease rates of temporal lobe neuropathy(TLN)[6,16]. A randomized control trial comparing QoL early stage NPC patients between 2D-RT with IMRT treatments found that IMRT treatment was superior in swallowing and speech problems after treatment[14]. However, another randomized control trial found no significant differences in the two treatments in terms of patient-reported xerostomia[6].

At present, there are two reviews on the efficacy of IMRT. Co J et al. included 3 RCTs focusing on partial oncologic outcomes at 1-year follow up and considered only xerostomia as the early and late effect parameter[17]. Only one RCT reported the oncology outcomes in the locally advanced stage of disease[12]. The other review combined 2D-RT and 3D-RT treatments, and found the potential advantages of IMRT treatment[18]. However, the disadvantage of marginal and geographic misses should be considered in IMRT treatment[19]. Recent data from numerous retrospective studies have demonstrated a potential survival benefit from IMRT in NPC patients[20–24]. Hence, it is necessary to compare the efficacy of IMRT and 2D-RT separately. Although there are many potential advantages for IMRT treatment to NPC patients, it is still unclear whether the dosimetric improvements can be translated into clinical significantly advantages. In light of these findings, we performed a systematic review and meta-analysis of the currently available evidences to further compare the clinical oncologic outcomes and potential toxicities of intensity-modulated radiation therapy (IMRT) with 2D-RT in NPC patients.

## Materials and methods

This systematic review was carried out in accordance with the Cochrane handbook, and the evidence was reported using the Preferred Reporting of Systematic Reviews and Meta-Analyses (PRISMA) guidelines[25,26] (Supporting Information, S1 Table). A prospective protocol including objectives, study selection, outcomes of interest and statistical analysis methods was also planned according to PRISMA guidelines.

### Search strategy and selection criteria

We search all the pertinent published and reported clinical trials up to December 1, 2018 through the following electronic databases: Pubmed, Cochrane Library, Medline and Embase; The medical subject headings and text words used include nasopharyngeal carcinoma, intensity-modulated radiation therapy and two-dimensional radiation therapy. Among them, the details retrieved in PubMed is as follows, "Nasopharyngeal Neoplasms" [Mesh] AND "Radiotherapy, Intensity-Modulated" [Mesh] AND ("conventional radiotherapy" [All Fields] OR "two-dimensional radiotherapy" [All Fields]) AND (("0001/01/01"[PDAT]: "2018/12/01" [PDAT]) AND "humans"[MeSH Terms]). Further details of the search strategy are shown in Supporting Information, S2 Table. The local publications were identified via manual searches in professional organizations and libraries. All studies included were in English.

Studies were included if the following criteria were satisfied: (1) types of studies: RCT, or retrospective study; (2) types of participants: participants were treated by radiotherapy either primarily or combined with surgery or chemotherapy (such as neoadjuvant, concurrent, or adjuvant); (3) types of interventions: compare IMRT alone with 2D-RT for NPC patients; (4) outcomes: reported 5-year overall survival (OS), progression-free survival (PFS), distant metastasis-free survival (DMFS), local relapse-free survival (LRFS) and late radiotoxicity. If different articles involved the same study population, the study with the complete or most recent survival data was included. Studies were excluded if they met any of the following criteria: (1) editorials, commentaries, letters, and case reports; (2) the survival data could not be extracted from the research.

#### Data extraction

Data extraction was conducted independently by two reviewers, and the disagreements were resolved in consultation with a third reviewer. The relevant characteristics extracted from each study included author, publication year, study design, number of IMRT and 2D-RT patients, tumor stage, the radiation dose, survival outcome, and late toxicities. Survival outcomes in present study mainly include 5-year overall survival (OS), progression-free survival (PFS), distant metastasis-free survival (DMFS), and local relapse-free survival (LRFS). These endpoints were defined as the data from the start of RT to the data of death from any cause (OS), the first observation of local or regional recurrence or distant metastasis (PFS), the first occurrence of distant metastasis (DMFS) and the first observation of local recurrence (LRFS). Where OS is the primary endpoint and the remaining indicators are the secondary endpoints.

#### Statistical analysis

Statistical analyses including combination of statistical pooling of data and narrative synthesis of the evidence were performed using STATA 14.0 (Stata Corporation, College Station, TX). All data analyses were expressed with odd ratios (OR) with 95% confidence intervals (CI). The results were not statistically significant if the 95% CI overlap 1, otherwise is statistically significant. For survival outcomes and quality of life, IMRT could bring favorable survival to the NPC patient when the OR is greater than 1. For the analysis of toxic effects, the incidence of toxic effects of NPC patients caused by IMRT treatment is less than 2D-RT when OR less is less than 1.

The heterogeneity between studies was quantified using the Q test[27] and the *I*-squared statistic[28], and heterogeneity was defined as *I*^2^ > 50% in *I*^2^ metric or *P* value < 0.10 in Q test. The meta-analysis was performed using a fixed effects model (the Mantel–Haenszel method) if the level of heterogeneity was acceptable (*P* > 0.10, or *P* ≤ 0.10 but *I*^2^ ≤ 50%), otherwise a random effects model was used for the meta-analysis. Subgroup analysis was used to explore the reasons for the existence of heterogeneity when heterogeneity exists, and sensitivity analysis is used to assess the stability of the results by excluding each study from the meta-analysis and comparing the point estimates including and excluding the study. Publication bias in the pooled analysis was examined using egger’s funnel plot[29], whereby asymmetries in the funnel plot showed publication bias. All *P* value < 0.05 was considered statistically significant.

## Results

### Search results and description of studies

**Fig 1:**
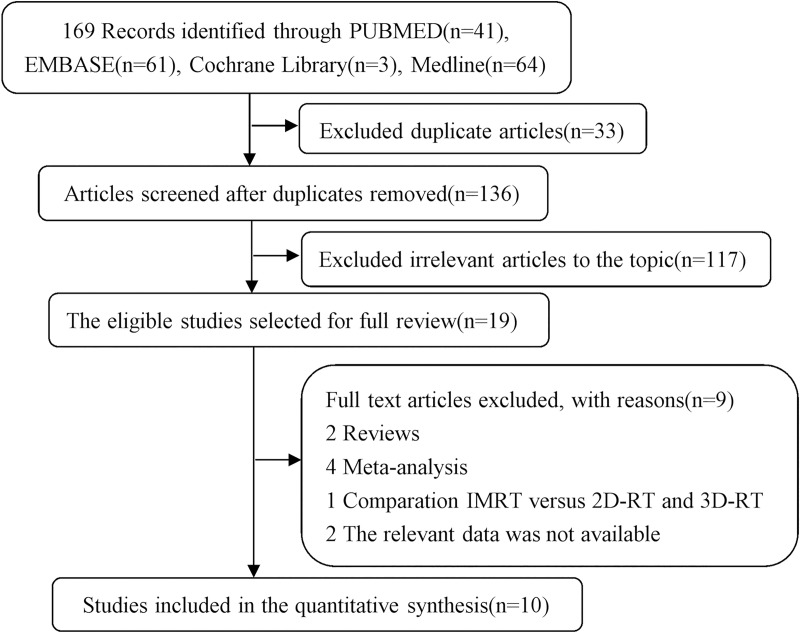
Process of identifcation and selection of relevant articles in this meta-analysis.

The search of literature on the effectiveness of IMRT versus 2D-RT for NPC yielded 169 references (Fig 1). Of these references, 33 duplicates were excluded after title review. By reviewing abstract or full-text, 117 irrelevant publications were excluded because these studies were one arm treatment studies and 3D radiotherapy studies. Full texts of 19 studies were then reviewed for eligibility. Of the 19 full articles retrieved, 1 was further excluded for comparison IMRT versus 2D-RT and 3D-RT, 2 for reviews, 2 for insufficient data, and 4 for meta-analysis. Finally, A total of 10 articles met the inclusion criteria and were entered into qualitative analysis[6,11,12,16,20–24,30].

The characteristics of the 10 studies are summarized in Table 1 and Table 2. We included 13304 patients in the meta-analysis, of whom 5212 received IMRT and 8092 were allocated to 2D-RT alone group. Stages I/II comprised 20.6% of the patients, and the remaining 79.4% were stage III/IV. Seven studies had evaluated the LRFS of NPC patients. DMFS data and late xerostomia data were available in 5 studies, and OS data and DMFS data in 6 studies. PFS data was available in 4 studies. Hearing loss data were in 2 studies, and TLN data and trismus data in 3 studies.

**Table 1:** Characteristics of the studies.

Author	Year	Country	Study design	The stage of patients	Treatment	No. of patients (n)	Male (%)	Median age	Stage III/IV, n (%)	T3-4, n(%)	N2-3, n(%)
Moon et al.	2016	Korean	Nonrandomized	T1-4N0-3M0	IMRT	497	346(69.6)	NR	378(76.1)	214(43.0)	295(59.4)
					2D-RT	350	260(74.3)	NR	269(76.8)	166(47.5)	187(53.4)
Kam et al.	2007	Hong Kong	Randomized	T1-2bN0-1M0	IMRT	28	21(75.0)	45.5	0	NR	NR
					2D-RT	28	19(68.0)	50.5	0	NR	NR
Lai et al.	2011	Guangzhou, China	Nonrandomized	M0	IMRT	512	393(76.8)	NR	344(67.2)	266(51.9)	169(33.0)
					2D-RT	764	566(74.1)	NR	532(69.6)	437(57.2)	232(30.4)
Peng et al.	2012	Wuhan, China	Randomized	M0	IMRT	306	221(72.2)	46.7	210(68.6)	NR	NR
					2D-RT	310	210(67.7)	44.8	212(68.4)	NR	NR
Qiu et al.	2017	Guangzhou, China	Nonrandomized	M0	IMRT	102	74(72.5)	NR	97(95.1)	92(90.2)	67(65.7)
					2D-RT	74	55(74.3)	NR	72(97.3)	70(94.6)	48(64.9)
Tang et al.	2015	Guangzhou, China	Nonrandomized	M0	IMRT	540	415(76.9)	44.5	NR	444(82.3)	NR
					2D-RT	512	380(74.2)	44.5	NR	382(74.6)	NR
Zhang et al.	2015	Guangzhou, China	Nonrandomized	M0	IMRT	2245	1495(66.6)	NR	1789(79.7)	1536(68.4)	902(40.2)
					2D-RT	4836	3582(74.1)	NR	3864(79.9)	3197(66.1)	1662(46.4)
Zhou et al.	2013	Guangzhou, China	Nonrandomized	M0	IMRT	506	NR	NR	NR	NR	NR
					2D-RT	747	NR	NR	NR	NR	NR
Zhong et al.	2013	Zhanjiang, China	Nonrandomized	T1-2bN0-2M0	IMRT	32	NR	NR	11(34.4)	NR	NR
					2D-RT	37	NR	NR	13(35.1)	NR	NR
Lee et al.	2014	Hong Kong	Nonrandomized	M0	IMRT	444	333(75.0)	52	408(92.0)	302(68.0)	377(85.0)
					2D-RT	434	312(72.0)	48	256(59.0)	161(37.0)	165(38.0)

n = number of patients; IMRT, intensity-modulated radiotherapy; 2D-RT, 2-dimensional conventional radiotherapy; NR, not report.

**Table 2:** Characteristics of the studies.

Author	Treatment	Chemotherapy, %				Surgery, %	RT dose of tumor, Gy
		Yes	Neoadjuvant	Concurrent	Adjuvant		
Moon et al.	IMRT	NR	30.0	82.3	31.8	NR	69.49(± 3.18)
	2D-RT	NR	54.0	30.0	14.0	NR	69.58 (±3.34)
Kam et al.	IMRT	NR	NR	0	NR	NR	66 ± BT
	2D-RT	NR	NR	0	NR	NR	66 ± BT
Lai et al.	IMRT	81.4^a^	NR	NR	NR	NR	60–64
	2D-RT	78.4 ^a^	NR	NR	NR	NR	68–76
Peng et al.	IMRT	NR	31.7	34.3	60.5	NR	74 ± BT
	2D-RT	NR	34.5	33.2	57.4	NR	70–74 ± BT
Qiu et al.	IMRT	NR	15.7	24.5	NR	NR	62–70
	2D-RT	NR	44.6	20.3	NR	NR	66–80
Tang et al.	IMRT	87.0 ^a^	NR	NR	NR	NR	68
	2D-RT	82.1 ^a^	NR	NR	NR	NR	68–76
Zhang et al.	IMRT	46.6	NR	NR	NR	NR	68
	2D-RT	54.0	NR	NR	NR	NR	68–76
Zhou et al.	IMRT	67.0	NR	NR	NR	NR	68
	2D-RT	43.6	NR	NR	NR	NR	68–76
Zhong et al.	IMRT	NR	NR	NR	NR	NR	70
	2D-RT	NR	NR	NR	NR	NR	70
Lee et al.	IMRT	NR	NR	3	NR	4	70
	2D-RT	NR	NR	2	NR	3	66

n, number of patients; IMRT, intensity-modulated radiotherapy; 2D-RT, 2-dimensional conventional radiotherapy; NR, not report.

^a^ Chemotherapy in stage III–IV patients

### Survival outcomes

**Fig 2:**
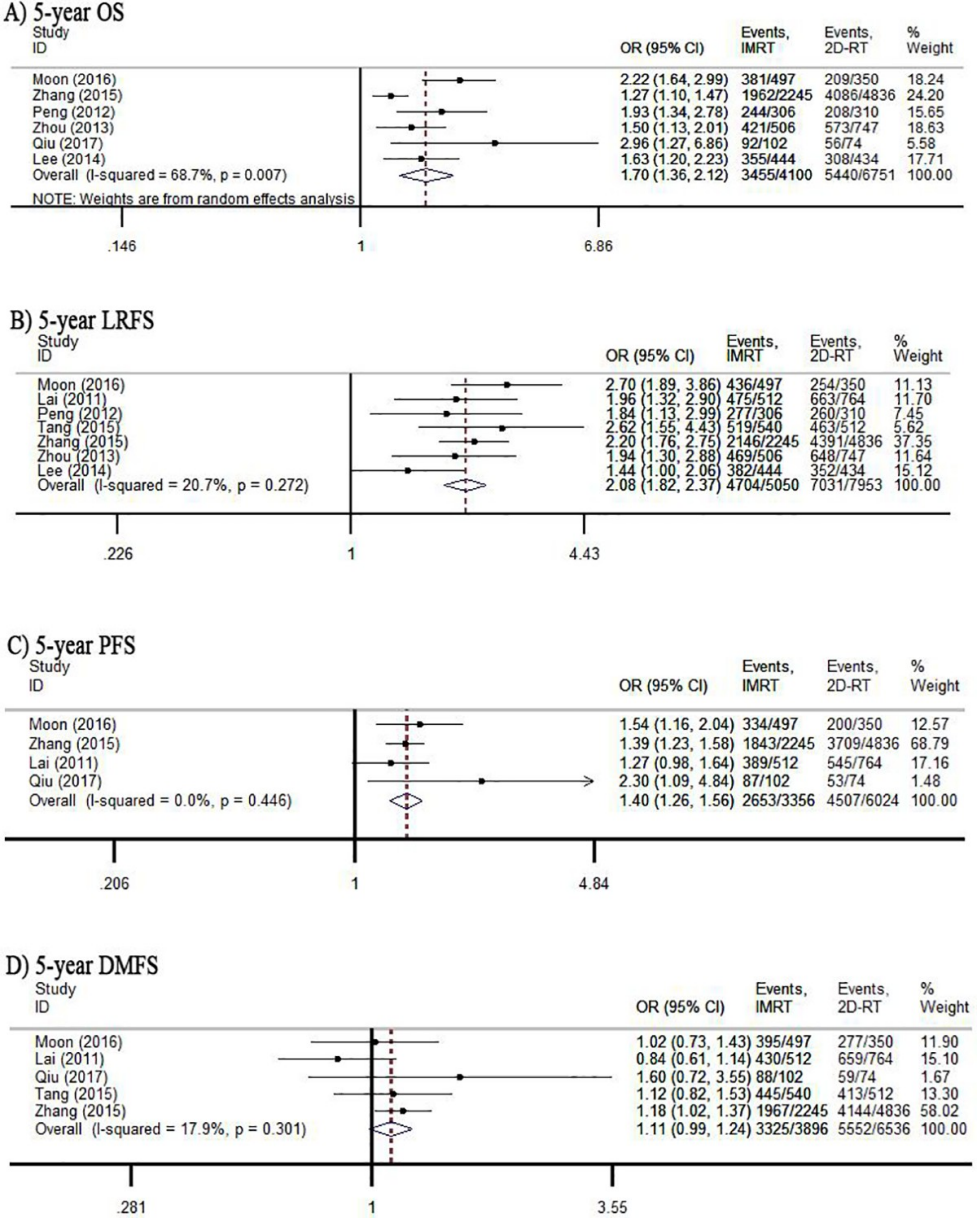
Forest plot of comparison between IMRT and 2D-RT for survival outcomes.

The IMRT group significantly improved 5-year OS in patients with NPC. The pooled OR and 95% CI for 5-year OS were 1.70 [1.36, 2.12]. As for 5-year LRFS, seven trials including 13003 patients were identified with outcome measurements. The pooled analysis showed that compared with 2D-RT, IMRT was associated with better 5-year LRFS (OR = 2.08, 95% CI:1.82–2.37). As for 5-year PFS, IMRT treatment was better than 2D-RT for NPC patients (OR = 1.40, 95% CI: 1.26–1.56). Six studies included in 5-year DMFS pooled analysis and heterogeneity was not found among these studies (*I*^2^ = 17.9%, *P* = 0.301). However, there was no difference between IMRT treatment and 2D-RT treatment for 5-year DMFS (OR = 1.11, 95% CI: 0.99–1.24) (Fig 2). When stratified into each tumor stage, there were no significant differences seen in terms of 5-year DMFS in NPC. The different effectiveness of two treatments for partial stage of patients was found in 5-year OS, 5-year LRFS, and 5-year PFS (all *P*< 0.05) (Supporting Information, S3 Table).

### Late toxicities

**Fig 3:**
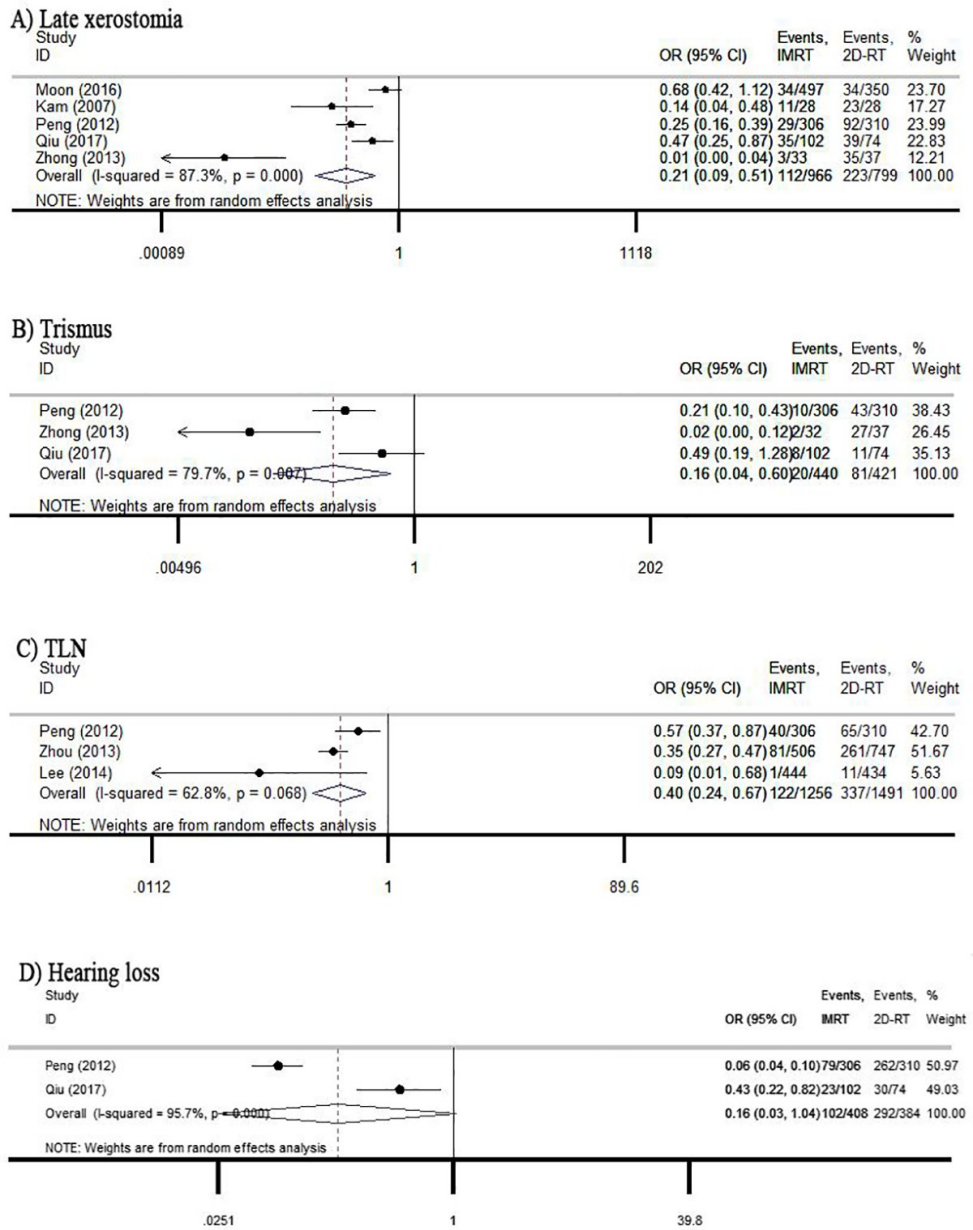
Forest plot of comparison between IMRT and 2D-RT for late toxicities.

Severe late xerostomia is a common problem in patients with head and neck cancer, especially 2D-RT treatment. Moreover, the radiation-induced chronic toxicity affected the survival of patients. Five studies reported late xerostomia and found that IMRT reduced the risk of the toxicity compared to 2D-RT treatment for NPC patients (OR = 0.21, 95% CI: 0.09–0.51) (Fig 3).

With regard to trismus and TLN, we retrieved relevant studies and two pooled analysis indicated that the incidence of trismus and temporal lobe neuropathy induced by radiation was significantly lower in IMRT group than in 2D-RT group (OR = 0.16; 95% CI: 0.04–0.60; OR = 0.40; 95% CI: 0.24–0.67) (Fig 3). We included two studies about hearing loss, and a meta analysis showed there was no significant difference between two treatment groups (OR = 0.16; 95% CI: 0.03–1.04).

### Quality of life (QoL)

By consulting the literature, we found two studies[14,31] that systematically assessed the quality of life of NPC patients through European Organization for Research and Treatment of Cancer Quality of Life Questionnaire-Core 30 (EORTC QOL-C30) and The EOTRC Quality of Life Questionnaire-Head and Neck 35 (EORTC QOL-H&N35). Pan et al. [31] showed that IMRT (n = 59) had higher mean scores in both functional and symptom scales of EORTC QLQ-C30 for stage II NPC patients than 2D-RT (n = 47). In addition, the study demonstrated that 2D-RT adversely affected patients with regard to global QoL, symptom scales, and functional scales compared with IMRT group (all *P* <0.001).

Pow et al.[14] showed that there were significant differences in scores between the two groups for the symptom item diarrhea at 2 months (*P* = 0.007) post-RT and for the functional scale role functional (revised) (*P* = 0.035) at 12 months after treatment, and the IMRT group had lower symptom item scores and higher functional scale scores demonstrating a better condition for NPC patients. IMRT group had lower scale scores in speech problem at 6 and 12 months post-RT and swallowing at 12 months aspects (*P* <0.05).

### Sensitivity analysis and publication bias

**Fig 4:**
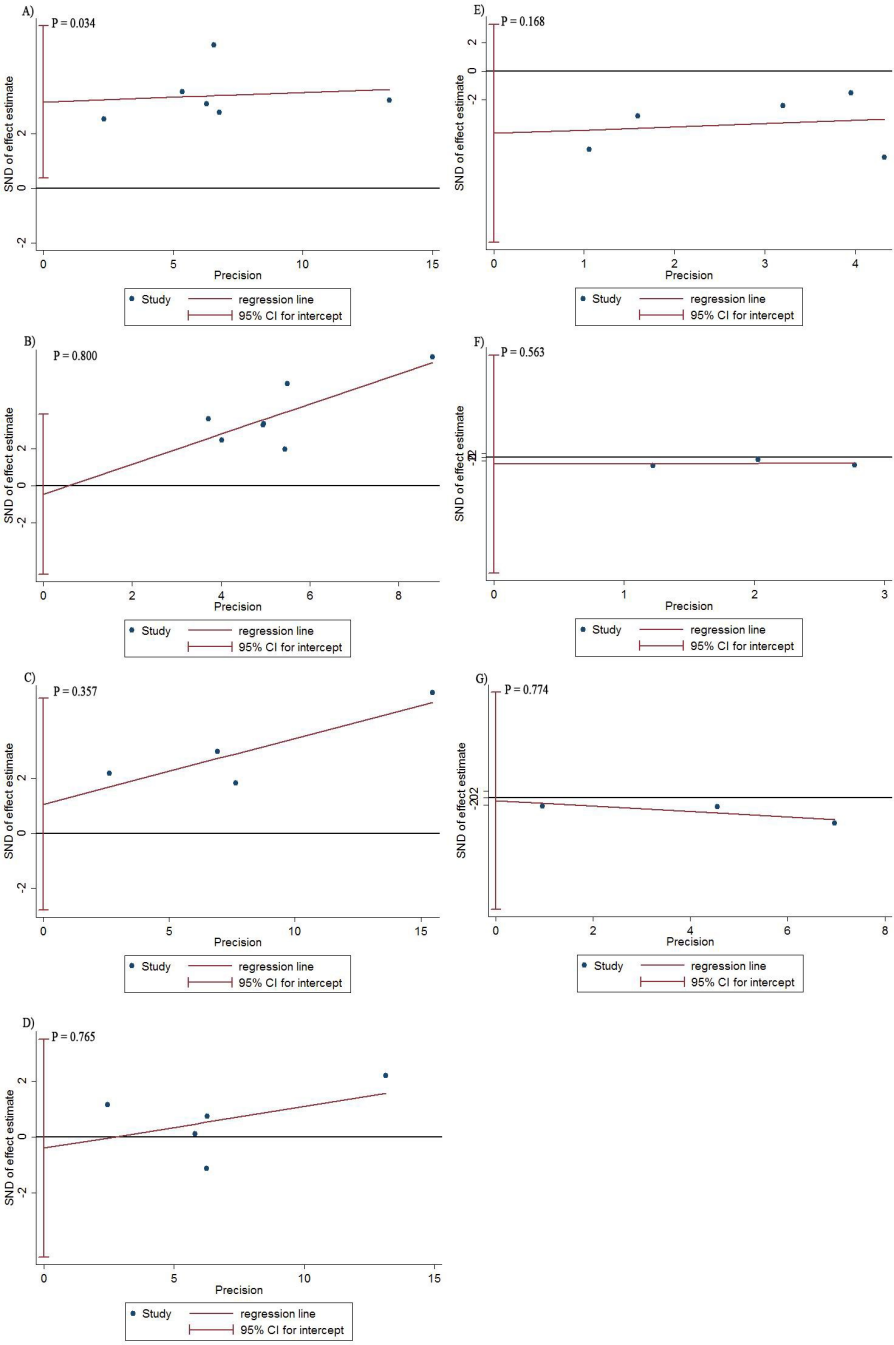
Funnel plots of publication bias summary for meta-analysis of 5-year OS (A), 5-year LRFS (B), 5-year PFS (C), 5-year DMFS (D), late xerostomia (E), trismus (F), and TLN (G).

Heterogeneity was not found in terms of 5-year LRFS, 5-year PFS, 5-year DFS, and 5-year DMFS in the chi-square and *I*-square tests, and a random effect model was used when the heterogeneity was found in pooled analysis. We used the egger’s funnel plot to assess the publication bias for evaluation of OS, LRFS, DMFS, late xerostomia, and trismus. The egger’s test and funnel of hearing loss was not conducted due to the number of included studies of them was less than three. The funnel plot showed no publication bias in LRFS (*P* = 0.800), PFS (*P* = 0.357), DMFS (*P* = 0.765), late xerostomia (*P* = 0.168), trismus (*P* = 0.563), and TLN (*P* = 0.774) (Fig 4). We found publication bias in OS by egger’s test (*P* = 0.034). The results of the sensitivity analysis are shown in the supplementary materials (Supporting Information, S1–S7 Figs).

## Discussion

Radiation therapy (RT) is widely adopted in the treatment of head and neck neoplasms, such as NPC. RT yields better conditions for patients in different sites when compared with surgery and thus is frequently used as conservative approaches. During the past decades, new RT technologies have emerged rapidly and the delivery of RT has evolved from two dimensional (2D-RT) techniques, based primarily on X-ray images and manual calculations[32], to IMRT recently, based on non-uniform radiation beam intensities to deliver an adequate dose to the target tumor while minimizing irradiation of normal tissue such as parotid, temporal lobe outside the target[33,34]. Therefore, it is vital to undertake a systematic comparison of clinical outcomes including overall survival, loco-regional control and late toxicities between IMRT treatment and 2D-RT treatment for NPC patients.

In present study, we found the effectiveness of IMRT treatment was superior to 2D-RT for NPC patients in 5-year OS (OR = 1.70; 95% CI: 1.36–2.12), 5-year LRFS (OR = 2.08; 95% CI: 1.82–2.37), and 5-year PFS (OR = 1.40; 95%CI: 1.26–1.56). There were five studies comparing the efficacy of IMRT and 2D-RT in patients with NPC in term of disease stage stratification[11,12,20,21,35]. Peng et al.[12] showed that oncologic outcomes of NPC patients with IMRT treatment were better than 2D-RT in T4 (*P* = 0.05), N2 (*P* = 0.026), and stage III (*P* = 0.018) disease. Lai et al.[11] found that IMRT increased the 5-year local progression-free survival rate only in T1 stage patients (*P* = 0.016). Both [35] and [20] found that patients with T3-4 had higher 5-year local progression-free survival rate when receiving IMRT(*P* = 0.022, *P* = 0.018, respectively). Moreover, the result LRFS and OS rates of patients receiving IMRT would increase at all stages disease also had been found in another study[21].

Regarding the toxicity or side effects caused by radiotherapy, it is the most noteworthy because it greatly affects the quality of life of patients. Although IMRT has an advantage to deliver an adequate dose on complex tumoral targets with dose-escalation while sparing surrounding normal organs at risk, such as salivary glands and brain stem. Previous reports showed that the use of IMRT for the treatment of NPC had higher local control rates and less late toxicity than with 2D-RT [6,8,36,37]. The present study combined the evidence to compare toxicity including late xerostomia, trismus and TLN (temporal lobe neuropathy) induced by radiation and found NPC patients receiving IMRT had fewer toxicities than 2D-RT. Among these toxic effects, xerostomia is most worthy of attention in the irradiation of the head and neck. Five studies including 1765 patients in our study demonstrated that IMRT has a significant effect in reducing the incidence of late xerostomia compared to 2D-RT (OR = 0.21; 95% CI: 0.09–0.51). Compared with the 2D-RT group, a significant overall benefit in favor of IMRT was found regarding stimulated parotid flow rate (SPFR) and stimulated whole saliva flow rate (SWSFR) in previous studies [6,14]. The improvements in the IMRT technique result in better overall survival and longer tumor control while the patient is receiving radiation therapy, but this can also increase the incidence of complications in the later stages. Trismus and TLN are common complications caused by irradiation in NPC patients. Trismus, greatly restricted mouth opening, is a common problem in head and neck neoplasm and is frequently reported in former literature [38]. The incidence of trismus varies greatly by different studies, and it rang from 5% to 38% [39,40]. Previous study demonstrated that trismus may reduce the mouth’s open level because of irradiation [41], thus might cause the nutritional deficiencies of patients. We have found IMRT reduced significantly the rate of trismus in this study (OR = 0.16; 95% CI: 0.04–0.60). There were some reports showed that the temporal lobe injury caused by irradiation has become a major factor in the death of more than half of patients [42,43]. Three studies included 2747 patients compared the radiation-induced TLN for patients with NPC, and our result demonstrated that IMRT group had significantly lower incidence of TLN occurrence compared with 2D-RT group. Additionally, the advantage that IMRT can minimize unnecessary doses to reduce risk of toxicities for the temporal lobes in patients with NPC was found in a dosimetry study [13].

Although previous reviews[17,18] have explored the differences in efficacy between the two treatments for NPC patients, there are several strengths for this study. Firstly, there is a lack of comprehensive review comparing the efficacy between IMRT with 2D-RT alone for NPC patients. Although Co J et al. conducted the first meta-analysis to compare the efficacy of the two modalities, the follow-up time was only one year and the conclusion of tumor outcomes came from one evidence. Secondly, in addition to the advantages of OS and LRFS found in previous study[18], we also found that IMRT was superior to 2D-RT in PFS, and no difference was found between the two treatments for DMFS. Distant metastasis remains a challenge in the treatment of NPC patients. Thirdly, since the current research is updated and the clinical evidences are inconsistency, we have merged these new studies to explore the difference of two treatments.

Present study is the first comprehensive synthesis of current evidences to compare the efficacy of IMRT and 2D-RT for patients with NPC, but there are still some limitations in the study. Firstly, present study included some non-random and retrospective studies. Inherent limitations mostly exist in observational studies, and unbalanced clinical factors and patients receiving other treatments or not inevitably result in heterogeneity, so as to affect study results. Secondly, since the lack of sufficient evidence, we did not assess the impact of chemotherapy or RT on oncology outcomes as individual studies reported them, and found the publication bias in OS. Finally, we reviewed the current literatures about the effectiveness after two treatments in different stage of NPC patients, but did not conduct stratified analysis of tumors.

## Conclusions

This study has identified 10 comparative studies of IMRT and 2D-RT. The meta-analysis demonstrates that IMRT provides improved long-term tumor overall survival and local control including LRFS and PFS. Additionally, IMRT yields a lower incidence of late toxicities induced by irradiation in NPC patients. Compared to 2D-RT, IMRT may be an effective treatment for patients with NPC. Further intensive studies should be pursued to examine the association.

## Supporting information

S1 Fig The sensitivity analysis of 5-year OS. (DOC)

S2 Fig The sensitivity analysis of 5-year LRFS. (DOC)

S3 Fig The sensitivity analysis of 5-year PFS. (DOC)

S4 Fig The sensitivity analysis of 5-year DMFS. (DOC)

S5 Fig The sensitivity analysis of 5-year late xerostomia. (DOC)

S6 Fig The sensitivity analysis of 5-year trismus. (DOC)

S7 Fig The sensitivity analysis of 5-year TLN. (DOC)

S1 Table The PRISMA checklist. (DOC)

S2 Table The Search strategy. (DOC)

S3 Table Literature review: Stratification analyses. (DOC)
