# Supplementary figures and images for: The effectiveness of intensity-modulated radiation therapy versus 2D-RT for the treatment of nasopharyngeal carcinoma: A systematic review and meta-analysis
Source: PLoS One. 2019 Jul 10;14(7):e0219611. doi: 10.1371/journal.pone.0219611 (PMC6619803; doi:10.1371/journal.pone.0219611)

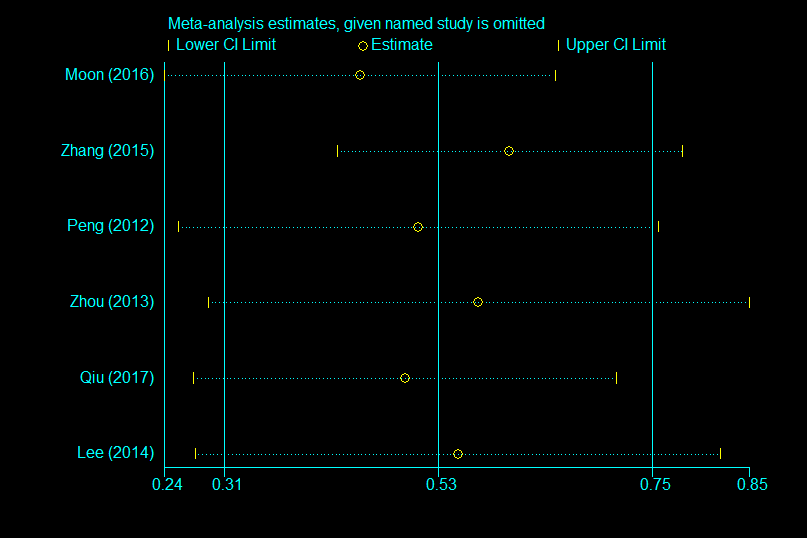


S1 Fig. The sensitivity analysis of 5-year OS

Supplement: S1 Fig — (DOC) [file pone.0219611.s001.doc]

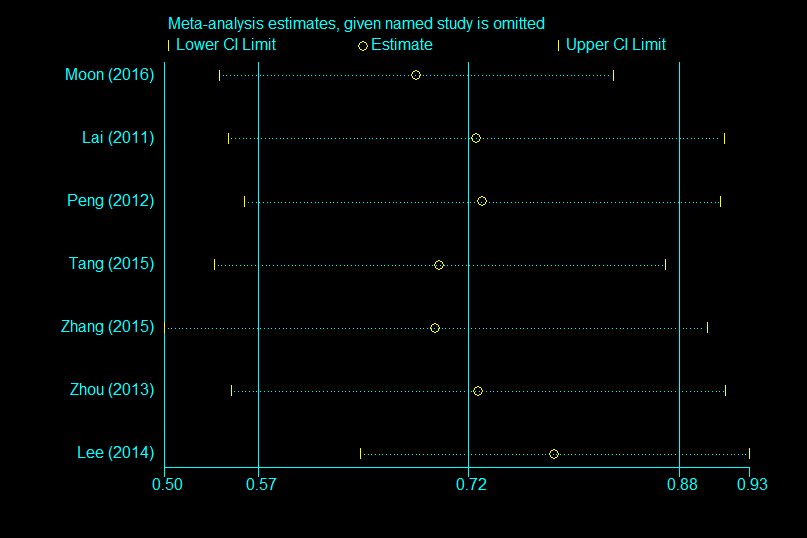


S2 Fig. The sensitivity analysis of 5-year LRFS

Supplement: S2 Fig — (DOC) [file pone.0219611.s002.doc]

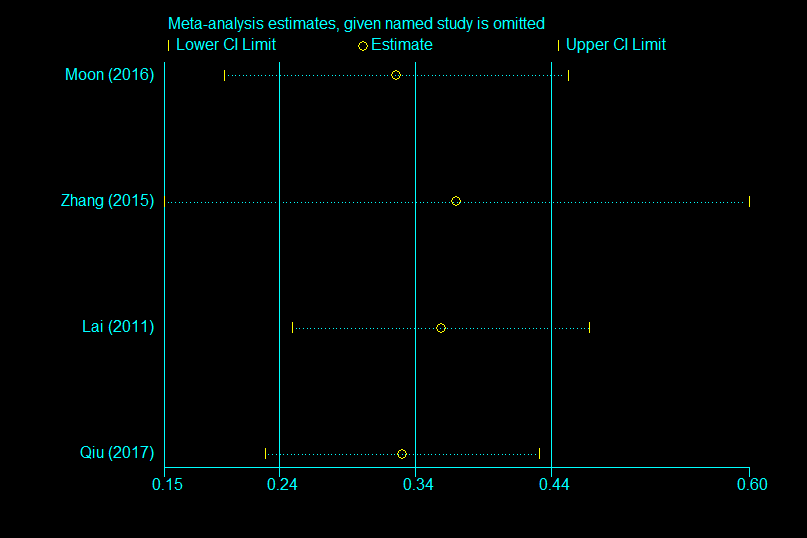


S3 Fig. The sensitivity analysis of 5-year PFS

Supplement: S3 Fig — (DOC) [file pone.0219611.s003.doc]

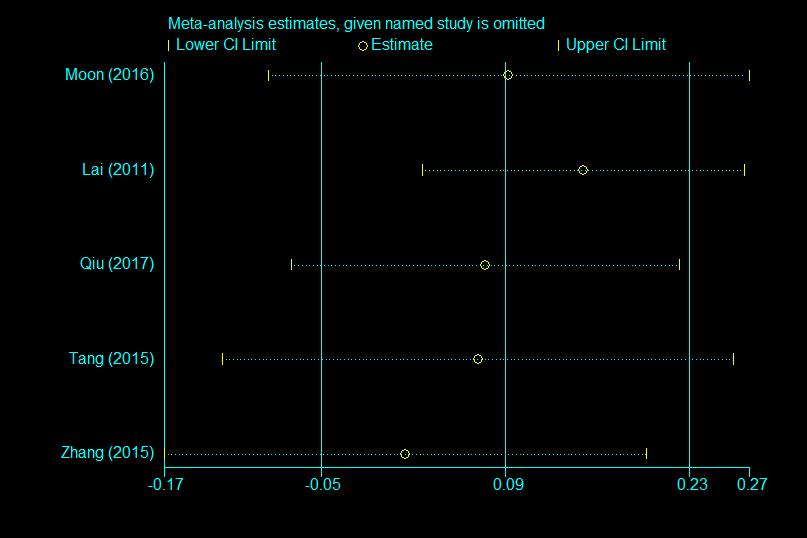


S4 Fig. The sensitivity analysis of 5-year DMFS

Supplement: S4 Fig — (DOC) [file pone.0219611.s004.doc]

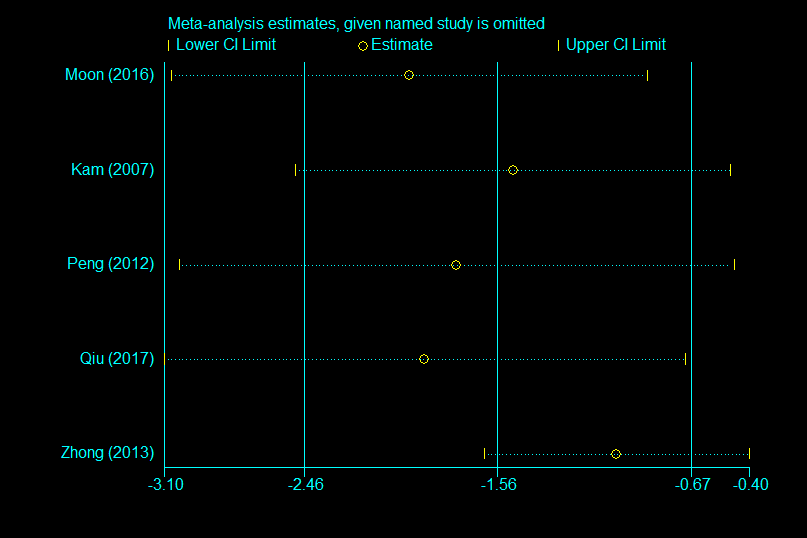


S5 Fig. The sensitivity analysis of 5-year late xerostomia

Supplement: S5 Fig — (DOC) [file pone.0219611.s005.doc]

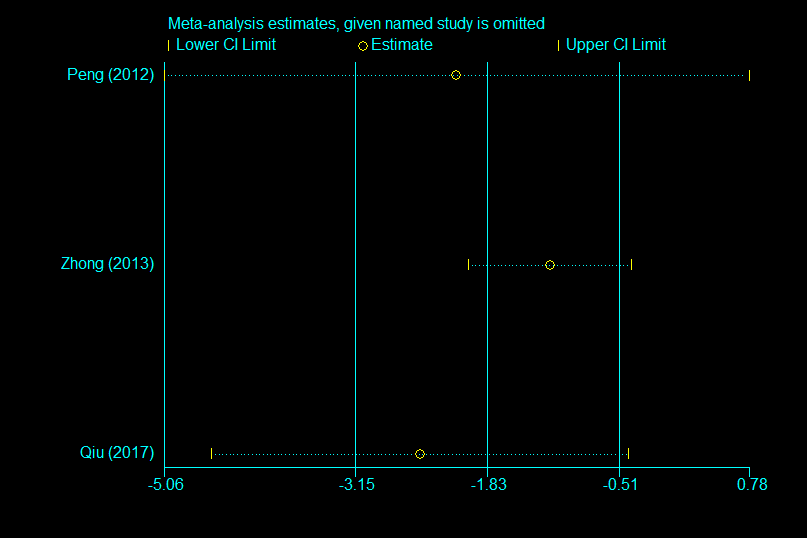


S6 Fig. The sensitivity analysis of 5-year trismus

Supplement: S6 Fig — (DOC) [file pone.0219611.s006.doc]

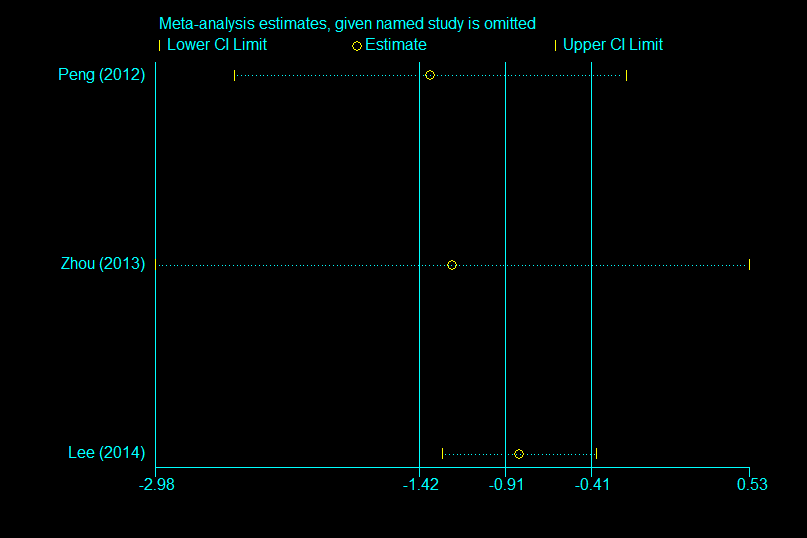


S7 Fig. The sensitivity analysis of 5-year TLN

Supplement: S7 Fig — (DOC) [file pone.0219611.s007.doc]
