# Supplementary material for: The effectiveness of intensity-modulated radiation therapy versus 2D-RT for the treatment of nasopharyngeal carcinoma: A systematic review and meta-analysis
Source: PLoS One. 2019 Jul 10;14(7):e0219611. doi: 10.1371/journal.pone.0219611 (PMC6619803; doi:10.1371/journal.pone.0219611)
Supplement: S2 Table — (DOC) [file pone.0219611.s009.doc]

**S2 Table. The Search strategy.**

**(The following are the retrieval strategies for two of these databases)**

**a:** Search strategy in PubMed

| # | Query |
| --- | --- |
| #1 | "Nasopharyngeal Neoplasms"[Mesh] |
| #2 | "Radiotherapy, Intensity-Modulated"[Mesh] |
| #3 | "conventional radiotherapy"[All Fields] OR "two-dimensional radiotherapy"[All Fields] |
| #4 | #1 AND #2 AND #3 |
| #5 | "0001/01/01"[PDAT] : "2018/12/01"[PDAT] |
| #6 | "humans"[MeSH Terms] |
| #7 | #4 NOT #5 AND #6 |

**b:** Search strategy in Embase

| # | Query |
| --- | --- |
| #1 | 'nasopharyngeal carcinoma':ab |
| #2 | 'intensity modulated radiation therapy':ab |
| #3 | 'conventional radiotherapy':ab OR 'two-dimensional radiotherapy':ab OR 'conventional two-dimensional radiotherapy':ab |
| #4 | #1 AND #2 AND #3 |
