# Supplementary material for: The effectiveness of intensity-modulated radiation therapy versus 2D-RT for the treatment of nasopharyngeal carcinoma: A systematic review and meta-analysis
Source: PLoS One. 2019 Jul 10;14(7):e0219611. doi: 10.1371/journal.pone.0219611 (PMC6619803; doi:10.1371/journal.pone.0219611)
Supplement: S3 Table — (DOC) [file pone.0219611.s010.doc]

S3 Table. Literature review: Stratification analyses.

| **Author** | **5-year oncology outcomes, 2D-RT vs. IMRT (*p*)** | | |  |  |
| --- | --- | --- | --- | --- | --- |
|  | **OS** | **LRFS** | **PFS** | **DMFS** | **RC** |
| Moon et al. |  | T1-2: 77.3% VS 92.3%(**<0.001**) |  |  |  |
|  |  | T3-4: 66.8% VS 81.4(**0.018**) |  |  |  |
| Kam et al. |  |  |  |  |  |
| Lai et al. |  | Total: 86.8% VS 92.7%(0.007) | Total: 71.4% VS 75.9%(0.088) | Total: 82.6% VS 84.0%(0.395) |  |
|  |  | T1: 94.4% VS 100%(**0.016**) | I: 93.6% VS 95.4%(0.898) | N0:90.7% VS 92.6%(0.285) |  |
|  |  | T2: 92.6% VS 95.3%(0.543) | II: 80.8% VS 88.3%(0.113) | N1: 83.6% VS 82.2%(0.771) |  |
|  |  | T3: 87.7% VS 89.8%(0.548) | III: 75.6% VS 76.5%(0.672) | N2: 75.1% VS 80.5%(0.311) |  |
|  |  | T4: 71.8% VS 82.1%(0.149) | IV: 53.1% VS 54.9%(0.743) | N3: 65.4% VS 60.9%(0.864) |  |
| Peng et al. | Total: 67.1% VS 79.6%(0.001) | Total: 83.8% VS 90.5%(0.046) |  |  | Total: 84.0% VS 91.7%(0.049) |
|  | II a: 85.8% VS 92.3%(0.648) | T2a: 94.7% VS 94.2%(0.962) |  |  | N1: 84.6% VS 90.2%(0.227) |
|  | II b: 77.7% VS 84.9%(0.359) | T2b: 88.4% VS 90.2%(0.850) |  |  | N2: 74.8% VS 93.9%(**0.026**) |
|  | III: 67.4% VS 79.9%(**0.018**) | T3: 80.6% VS 91.0%(0.122) |  |  | N3: 89.5% VS 91.6%(0.844) |
|  | IV a: 55.2% VS 72.9%(0.057) | T4: 62.2% VS 81.5%(**0.050**) |  |  |  |
|  | IV b: 45.6% VS 42.8%(0.586) |  |  |  |  |
| Qiu et al. |  |  |  |  |  |
| Tang et al. |  |  |  |  |  |
| Zhang et al. | Total: 84.5% VS 87.4%(<0.001) | Total: 90.8% VS 95.6%(<0.001) | Total: 76.7% VS 82.1%(<0.001) | Total: 85.7% VS 87.6%(0.056) |  |
|  | I: 98.4% VS 96.3%(0.985) | T1: 93.3% VS 97.6%(**0.045**) | I: 92.8% VS 97.5%(**0.025**) | N0:93.8% VS 94.9%(0.204) |  |
|  | II: 92.8% VS 95.1%(**0.021**) | T2: 90.2% VS 95.8%(**0.001**) | II: 82.1% VS 87.7%(**0.008**) | N1: 87.2% VS 89.5%(0.286) |  |
|  | III: 85.4% VS 88.3%(**0.025**) | T3: 91.9% VS 96.3%(**<0.001**) | III: 77.8% VS 81.7%(**0.009**) | N2: 81.9% VS 82.7%(0.652) |  |
|  | IV: 74.8% VS 78.6%(0.106) | T4: 87.5% VS 91.8%(**0.015**) | IV: 68.7% VS 76.4%(**0.015**) | N3: 76.7% VS 76.9%(0.680) |  |
| Zhou et al. |  |  |  |  |  |
| Zhong et al. |  |  |  |  |  |
| Lee et al. | I- II: 87.0% VS 91.0%(0.750) | T1-2:85.0% VS 92.0%(0.160) |  |  |  |
|  | III- IV:60.0% VS 79.0%(**<0.001**) | T3-4:74.0% VS 83.0%(**0.022**) |  |  |  |
